# The C-Terminal Effector Domain of Non-Structural Protein 1 of Influenza A Virus Blocks IFN-β Production by Targeting TNF Receptor-Associated Factor 3

**DOI:** 10.3389/fimmu.2017.00779

**Published:** 2017-07-03

**Authors:** Wei Qian, Xiaoqin Wei, Kelei Guo, Yongtao Li, Xian Lin, Zhong Zou, Hongbo Zhou, Meilin Jin

**Affiliations:** ^1^State Key Laboratory of Agricultural Microbiology, Huazhong Agricultural University, Wuhan, China; ^2^Department of Preventive Veterinary Medicine, College of Animal Science & Medicine, Huazhong Agricultural University, Wuhan, China; ^3^College of Agricultural and Animal Husbandry, Tibet University, Linzhi, China; ^4^College of Animal Husbandry & Veterinary Science, Henan Agricultural University, Zhengzhou, China; ^5^Key Laboratory of Development of Veterinary Diagnostic Products, Ministry of Agriculture, College of Veterinary Medicine, Huazhong Agricultural University, Wuhan, China

**Keywords:** influenza virus, non-structural protein 1, IFN-β, TNF receptor-associated factor 3, immune evasion

## Abstract

Influenza A virus non-structural protein 1 (NS1) antagonizes interferon response through diverse strategies, particularly by inhibiting the activation of interferon regulatory factor 3 (IRF3) and IFN-β transcription. However, the underlying mechanisms used by the NS1 C-terminal effector domain (ED) to inhibit the activation of IFN-β pathway are not well understood. In this study, we used influenza virus subtype of H5N1 to demonstrate that the NS1 C-terminal ED but not the N-terminal RNA-binding domain, binds TNF receptor-associated factor 3 (TRAF3). This results in an attenuation of the type I IFN signaling pathway. We found that the NS1 C-terminal ED (named NS1/126-225) inhibits the active caspase activation and recruitment domain-containing form of RIG-I [RIG-I(N)]-induced IFN-β reporter activity, the phosphorylation of IRF3, and the induction of IFN-β. Further analysis showed that NS1/126-225 binds to TRAF3 through the TRAF domain, subsequently decreasing TRAF3 K63-linked ubiquitination. NS1/126-225 binding also disrupted the formation of the mitochondrial antiviral signaling (MAVS)–TRAF3 complex, increasing the recruitment of IKKε to MAVS; ultimately shutting down the RIG-I(N)-mediated signal transduction and cellular antiviral responses. This attenuation of cellular antiviral responses leads to evasion of the innate immune response. Taken together, our findings offer an important insight into the interplay between the influenza virus and host innate immunity.

## Introduction

Retinoic acid-inducible gene I (RIG-I) is an RNA helicase that acts as a major host sensor for virus infections in the cytoplasm (1, 2). Influenza A virus (IAV) infection of cells is detected by RIG-I, which recognizes and binds to the 5′-ppp of the double-stranded RNA structure found within the panhandle of the IAV genome (3, 4). Next, upon binding of viral RNA, RIG-I is activated and undergoes a conformational change mediated by ATPase/Helicase activity, resulting in the exposure of caspase activation and recruitment domains (CARD). As the cascade continues, different residues in RIG-I are ubiquitinated by the E3 ligases TRIM25 (5) and RIPLET (6), resulting in RIG-I oligomerization. Subsequently, RIG-I interacts with the adaptor protein mitochondrial antiviral signaling (MAVS) *via* their CARD–CARD association, which in turn results in MAVS oligomerization. Through TNF receptor-associated factor 3 (TRAF3) or TRAF6, this leads to activation of the kinase complexes containing TBK1 or IKKε and IKKα/β/γ. Through several final phosphorylation steps, these kinases ultimately elicit antiviral and pro-inflammatory responses through interferon regulatory factor 3 (IRF3) and nuclear factor κB (NF-κB), respectively (7, 8).

Influenza A virus belongs to the orthomyxovirus family, containing eight negative-sense RNA segments in an enveloped viral particle encoding 14 or 17 proteins (9). This array of proteins contributes to virulence; including the proteins associated with viral RNA-dependent RNA polymerase (10) and the non-structural protein 1 (NS1). NS1 consists of 215-237 amino acids and comprises two functional domains: an N-terminal RNA-binding domain (RBD) (AA1 to 73) and a C-terminal effector domain (ED) (AA74-end) (11). The NS1 protein plays a crucial role in regulating the host antiviral response through various mechanisms. One important function of the NS1 protein involves inhibition of IFN production. The mechanism of this inhibition includes activation of the transcription factors IRF3 (12), NF-κB (13), and AP-1 (14), thus blocking IFN production. This efficient inhibitory effect is associated with an RIG-I signaling pathway through the NS1-RIG-I complex (15–17). Previous studies have indicated that NS1 is also related to two positive factors of RIG-I, the E3 ligases TRIM25 (18) and RIPLET (19). The residues E96/E97 of NS1 mediate their interaction with the coiled-coil domain of TRIM25, thus blocking both TRIM25 multimerization and RIG-I CARD domain ubiquitination. This subsequently induces lower levels of IFN-β (18). NS1 can also interact with RIPLET preventing the activation of RIG-I, although E96/E97 are not involved in that inhibition (19). The dsRNA binding ability of NS1 could also be playing a role in the pre-transcriptional inhibition of the interferon pathway by sequestering the pathogen-associated molecular patterns (PAMPs) that RIG-I recognizes. Two residues, R38 and K41, are required for the dsRNA binding activity of NS1 (20), thus highly impairing its ability to block interferon production.

In another similar pathway, NS1 has been shown to inhibit host mRNA synthesis by binding a cellular 3′ end-processing factor, the 30 kDa subunit of the cleavage and polyadenylation specificity factor (CPSF30), thus attenuating type I interferon (IFN-α/β) and other interferon stimulated gene (ISG) mRNAs that are involved in the antiviral response (21). The NS1 proteins encoded by the seasonal H1N1, H2N2, H3N2, and avian H5N1 viral subtypes strongly bind to CPSF30 (22), whereas PR8, 2009 pandemic H1N1, and novel H7N9 virus do not efficiently bind CPSF30 (23). It is noteworthy that cells infected with viruses expressing NS1 proteins in seasonal H3N2 and H2N2 viruses do not inhibit IRF3 activation. However, activation is blocked in cells infected with viruses expressing NS1 proteins in some, but not all, seasonal H1N1 viruses, 2009 pandemic H1N1, and avian H5N1 viruses. TRIM25 was previously reported to interact with each of these NS1 proteins, whether or not they block IRF3 activation, indicating that binding of TRIM25 by the NS1 protein does not necessarily lead to blocking of IRF3 activation (22). Hence, binding of the NS1 protein to dsRNA, RIG-I, and TRIM25 has not established that these NS1 interactions are responsible for inhibiting the activation of IRF3 and IFN transcription. In this case, one or more host factors may participate in the NS1 blocking of IRF3 activation.

In view of several yet undetermined roles of NS1 in the inhibition of interferon, we conducted a study aimed to determine the importance of the function of the N- and/or C-terminal domains of the NS1 protein in immune evasion. By using the luciferase reporter assay, we were able to demonstrate that the C-terminal ED (AA126 to 225, named NS1/126-225) of the NS1 protein was sufficient to inhibit the production of IFN-β driven by RIG-I(N). Mechanistically, NS1/126-225 was found specifically to interact with TRAF3, to dissociate MAVS–TRAF3 complex, and to decrease K63-linked polyubiquitination of TRAF3. This was shown to result in reduced IRF3-dependent production of IFN-β, with subsequent enhancement of virus replication. These data reveal a novel mechanism for how the influenza A virus NS1 protein induces inhibition of the host IFN production and may provide a potential target for antiviral drug development.

## Materials and Methods

### Viruses and Cells

The HPAI H5N1 virus strain, A/duck/Hubei/hangmei01/2006 (H5N1; designated H5N1/HM) was isolated from a duck. Influenza A virus (strain A/Puerto Rico/8/1934 H1N1), A/PR/8/34, was grown in our laboratories and stored until use. rNS1-SD30 was constructed as previously described (24). Influenza virus stocks of H5N1/HM, PR8, and rNS1-SD30 strains were amplified using 10-day-old embryonic chicken eggs and then titrated by determining log10 TCID50/ml values in MDCK cells. All cell experiments with H5N1 virus were performed in an Animal Biosafety Level 3 laboratory (BSL-3). This study was carried out in accordance with the recommendations of BSL-3, Huazhong Agricultural University (HZAU). The protocol was approved by the BSL-3 of HZAU. The recombinant vesicular stomatitis virus (VSV) encoding green fluorescence protein (VSV-GFP) was a gift from the Harbin Veterinary Research Institute (Harbin, China). Sendai virus (Sev) was grown in 10-day-old embryonic chicken eggs and titrated using a hemagglutination assay as previously described (25).

Human embryonic kidney 293T cells and HeLa cells were purchased from ATCC (Manassas, VA, USA) and cultured at 37°C with 5% CO_2_ in Roswell Park Memorial Institute-1640 medium (HyClone, China) supplemented with 10% fetal bovine serum (FBS) (PAN-Biotech, Germany), containing 100 U/ml penicillin, and 100 mg/ml streptomycin (GNM15140). Human lung epithelial cells (A549) were propagated in F12 medium (HyClone, China) with 10% FBS. MDCK cells were obtained from ATCC and propagated in Dulbecco’s Modified Eagle’s Medium (HyClone, China), maintained with 10% FBS.

### Reagents and Antibodies

Reagents were from the following suppliers: Lipofectamine 2000 (Invitrogen, USA); Poly(I:C) (Sigma, USA). Commercial antibodies were from the following suppliers: mouse monoclonal anti-Flag-tag antibody (Sigma, USA); mouse monoclonal and rabbit polyclonal anti-Hemagglutinin (HA)-tag antibodies, mouse monoclonal anti-gapdh and anti-β-actin antibodies (PMKbio, China); anti-MYC-tag mouse monoclonal and anti-TRAF3 rabbit polyclonal antibodies (Proteintech, USA); anti-Influenza A NS1 mouse monoclonal antibody (Santa Cruz Biotechnology, USA); anti-Influenza A NP (nucleoprotein) rabbit polyclonal antibody (GeneTex, USA); anti-Influenza A HA rabbit polyclonal antibody (Sino Biological, China); anti-IRF3 polyclonal and phospho-IRF3 (S396) polyclonal antibodies (EMD Millipore, USA); anti-Lamin A/C polyclonal antibody (Abclonal, USA); Cy3-conjugated goat anti-rabbit IgG and fluorescein isothiocyanate (FITC)-conjugated goat anti-mouse IgG (Invitrogen), and diamidino-2-phenylindole (DAPI) (100 ng/ml, Invitrogen).

### Plasmids

A plasmid encoding H5N1/HM NS1 with an N-terminal HA tag was amplified from viral cDNA (prepared from total RNA extracted from A549 cells after HM infection at 6 h) by reverse transcription-PCR. Plasmids were cloned into the ClaI and XhoI (TaKaRa, China) sites of the PCAGGS-HA (PCA) vector, and named HA-NS1. Using the same methods, PCA plasmids encoding PR8 and H9N2/W1 NS1 were constructed. PCA plasmids encoding H7N9/SH NS1 were constructed by synthesizing gene sequences from the Influenza A virus [A/Shanghai/02/2013(H7N9)] and subcloned into the PCA vector. PCA expression plasmids encoding truncated NS1 fragments, including HA-NS1/1-73, NS1/1-125, NS1/74-225, and NS1/126-225, were obtained by PCR amplification of HA-NS1. Plasmids expressing Flag-tagged RIG-I, the active CARD domain containing form of RIG-I [RIG-I(N)], MAVS, TBK1, TRAF3, IKKε, and pUb-HA were kindly provided by Dr. Zheng-fan Jiang (Peking University). Flag-TRAF6, luciferase reporter plasmid containing the IFN-β-promoter (IFN-β-Luc), and Renilla control plasmids pRL-TK were a gift from Dr. Ping Qian (Huazhong Agricultural University). The constitutively active IRF3, named IRF3(5D), was a kind gift from Dr. Yi-ling Lin (National Defense Medical Center). pUb-k48-HA and pUb-k63-HA were kindly provided by Dr. Hong-Bing Shu (Wuhan University). Plasmids expressing Myc-tagged RIG-I(N) were amplified from Flag-RIG-I(N) and cloned into pCMV-C-Myc vectors. Truncated forms of TRAF3 (AA1 to 346 and 347 to 568) with Flag tag were amplified from the full-length template and cloned into the p3FLAG-CMV-14 vector (provided by Dr. Zheng-fan Jiang). Plasmids expressing HA-tagged full-length MAVS was amplified by PCR and cloned into PCA vectors. All constructs were verified by DNA sequencing (Sangon Biotech Co., Ltd., Shanghai, China). The PCR primers used in this study are summarized in Table 1.

**Table 1:** Primers used in this study.

Primer name	Purpose	Sequence of oligonucleotide (5′–3′)	Accession no.
Non-structural protein 1 (NS1)	Full length	F ccatcgatATGGATTCCAACACTGTGTCA	EU594353
	Cloning	R ccctcgagTCAAACTTCTGACTCAATTGTTCT	
NS1/1-73	Truncated	F ccatcgatATGGATTCCAACACTGTGTCA	
	Cloning	R ccctcgagCTAATCCATTATTGCCTGGTC	
NS1/74-225	Truncated	F ccatcgatAAAACCATCATATTGAAAGCA	
	Cloning	R ccctcgagTCAAACTTCTGACTCAATTGTTCT	
NS1/1-125	Truncated	F ccatcgatATGGATTCCAACACTGTGTCA	
	Cloning	R ccctcgagCTAAGACTCCTCCTCCAGAATC	
NS1/126-225	Truncated	F ccatcgatGATGAGGCACTTAAAATGCC	
	Cloning	R ccctcgagTCAAACTTCTGACTCAATTGTTCT	
NS1/PR8	Full length	F ccatcgatATGGATCCAAACACTGTGTCA	NC_002020
	Cloning	R ccctcgagTCAAACTTCTGACCTAATTGTTC	
NS1/W1	Full length	F ccatcgatATGGATCCAAACACTGTGTCA	DQ465404
	Cloning	R ccctcgagCTATTTTGGAGAGAGTGGAGG	
NS1/SH	Full length	F ccatcgatATGGATTCCAATACTGTGTCA	KF021601
	Cloning	R ccctcgagCTACTTTGTAGAGAGTGGAGATCTC	
TNF receptor-associated factor 3 (TRAF3)-TD	Truncated	F cccaagcttATGGCAGACAGCATGAAGAG	NM_145725
	Cloning	R cgggatccGGGATCGGGCAGATCCGA	
TRAF3ΔTD	Truncated	F cccaagcttATGGAGTCGAGTAAAAAGATGG	
	Cloning	R cgggatccTTCCTCCCAGTTCTGCCG	
Mitochondrial antiviral signaling	Full length	F ggggtaccATGCCGTTTGCTGAAGACA	NM_020746
	Cloning	R ccctcgagCTAGTGCAGACGCCGCC	
IFN-β	Quantitative real-time PCR (qRT-PCR)	F GACGCCGCATTGACCATCTA	NM_002176
		R CCTTAGGATTTCCACTCTGACT	
β-Actin	qRT-PCR	F TGGACTTCGAGCAAGAGATGG	NM_001101
		R GGAAGGAAGGCTGGAAGAGTG	
PKR	qRT-PCR	F AAAGCGAACAAGGAGTAAG	NM_002759
		R GATGATGCCATCCCGTAG	
OASL	qRT-PCR	F AAGGTAGTCAAGGTGGGCTC	NM_003733
		R GGACTCTCTGCTCCATCCTC	
Mx1	qRT-PCR	F ACCACAGAGGCTCTCAGCAT	NM_001144925
		R CTCAGCTGGTCCTGGATCTC	
NP (HM)	qRT-PCR	F GCGTTCAGCCCACTTTCTCG	EU594350
		R GGGTTCGTTGCCTTTTCGTC	
NP (PR8)	qRT-PCR	F CCCAGGATGTGCTCTCTGATG	KC815515
		R TTCGTCCATTCTCACCCCTC	

### Luciferase Reporter Assays

293T cells seeded into 24-well plates were transiently transfected with plasmids encoding IFN-β-Luc and the internal control pRL-TK, together with other plasmids, as indicated. Cells were lysed with 1× passive lysis buffer, made by 1 volume of 5× passive lysis buffer (Promega) with 4 volumes of distilled water, followed by analysis of cell lysates for luciferase activity with a Dual-Luciferase Reporter Assay System kit (Promega) according to manufacturer’s instructions. All experiments were performed in triplicate and repeated at least three times. Data shown are mean ± SD from one representative experiment.

### ELISA for IFN-β

Culture supernatants were collected and centrifuged at 300 g for 10 min at 4°C to remove cell debris. Then 100 µl of the cleared medium or the IFN-β standard was used in duplicate for detection of IFN-β using a human IFN-β ELISA Kit (Elabscience, E-EL-H0085c), according to the manufacturer’s instructions.

### Immunoblot Analysis and Co-Immunoprecipitation (Co-IP)

Cellular lysates for detection of IRF3 dimerization by western blotting were prepared using non-reducing RIPA lysis buffer with 0.1% SDS containing protease and phosphatase inhibitors (Servicebio, China). For immunoblot experiments, treated cells were lysed in lysis buffer (10 mM phosphate pH 7.4, 137 mM NaCl, 1% NP-40, 0.5% sodium deoxycholate, and 0.1% SDS) supplemented with a protease inhibitor and phosphatase inhibitor cocktail. Total or fractionated cellular extracts were resolved on 10–12% SDS-PAGE. For Co-IP, treated cells were lysed in IP buffer (20 mM Tris-HCl pH 7.5, 150 mM NaCl, 1 mM EDTA, 1% NP-40, 10% glycerol, and protease/phosphatase inhibitor cocktail). The cell lysates were then incubated with anti-Flag or anti-HA antibodies rocking for 8 h at 4°C and the complexes were captured using Protein A/G PLUS-Agarose (Santa Cruz Biotech). Cell lysates and the immunoprecipitates were resolved by 10–12% SDS-PAGE and transferred to pure nitrocellulose membranes (GE). The membranes were blocked in 1% bovine serum albumin (BSA) in TBST buffer for 1 h at room temperature and probed with indicated primary antibodies for 1–2 h at room temperature. After hybridizing with either goat anti-rabbit or goat anti-mouse secondary antibodies at a dilution of 1:10,000 in TBST buffer, the membranes were washed with TBST buffer for four times (10 min each) before visualized with ECL reagents (Advansta).

### Immunofluorescence Assay

HeLa cells were plated onto coverslips in 24-well plates and transfected with the indicated plasmids. At 24 h post transfection, cells were washed once with phosphate-buffered saline (PBS) and fixed in 4% paraformaldehyde for 15 min. Cells were permeabilized with 0.1% Triton X-100 for 15 min and blocked for 1 h at room temperature with 1% BSA in PBS, followed by incubation with primary antibody for 1 h. After three washes with PBS containing 0.1% Tween 20, cells were incubated with FITC or Cy3-conjugated secondary antibodies for 1 h at room temperature and then incubated with 4′,6-DAPI for 10 min. Finally, the coverslips were washed extensively and fixed onto slides. Images were taken under a Zeiss LSM510 Meta confocal microscope (Carl Zeiss, Zena, Germany).

### Quantitative Real-time PCR (qRT-PCR)

Total RNA was isolated from cells using TRIzol reagent (Invitrogen) following manufacturer’s instructions and cDNA was prepared by using avian myeloblastosis virus reverse transcriptase (TaKaRa). cDNA was used for quantification of the indicated mRNA copy number on an ABI ViiA 7 PCR system (Applied Biosystems, USA) by using SYBR Green Master Mix (Rox). To detect and validate the specific amplification of PCR products, dissociation curve analysis of the products was conducted at the end of each PCR. Transcript levels of each gene were normalized with the expression of β-actin, and the $2^{-\Delta \Delta C_{t}}$ method was used to analyze gene expression in the samples (26). The primers used in qRT-PCR are listed in Table 1.

### RNA Interference

Three siRNA oligonucleotides against TRAF3 and the corresponding negative control siRNA were obtained from GenePharma. Sequences are as follows: si-1, 5′-CCACUGGAGAGAUGAAUAU-3′; si-2, 5′-GUUGUGCAGAGCAGUUAAU-3′; and si-3, 5′-CUGGUUACUUUGGCUAUAA-3′. Transfection of siRNA into 293T cells was performed by Lipofectamine 2000 according to manufacturer’s instructions.

### *In Vitro* Ubiquitination Assay

293T cells were seeded into 60-mm dishes and transiently transfected with the indicated plasmids. Thirty-six hours after transfection, cells were harvested and the lysates were prepared in a 1% NP-40 lysis buffer supplemented with a commercially available 0.1% protease inhibitor cocktail and a 10 mM deubiquitinase inhibitor N-ethylmaleimide (Sigma-Aldrich). Samples were immunoprecipitated with 1 µg anti-Flag antibodies along with 30 µl Protein A/G PLUS-Agarose. Polyubiquitination was detected using anti-HA antibodies.

### Influenza A Virus Infection of A549 Cells

A549 cells were transfected with the indicated plasmids for 24 h at 37°C. Cells were washed twice with F12 medium and then infected with H5N1/HM or PR8 at an indicated MOI (2 or 0.001, 0.01, and 0.1). After 1 h viral adsorption at 37°C, the inoculum was replaced with fresh medium and the cells were incubated at 37°C. Cells and supernatants were harvested at the selected time points for Co-IP experiments or virus titer measures, viral NP mRNA, and protein analysis.

### Statistical Analysis

The results are expressed as means ± SD. Statistical analyses were performed on data from triplicate experiments by using two-tailed Student’s *t*-test. A *P*-value of less than 0.05 was considered significant and a *P*-value of less than 0.01 was considered highly significant.

## Results

### The H5N1 NS1 Protein Inhibits the RIG-I(N)-Mediated Activation of IFN-β *via* Its C-Terminal ED in an RNA Binding-Independent Manner

**Figure 1:**
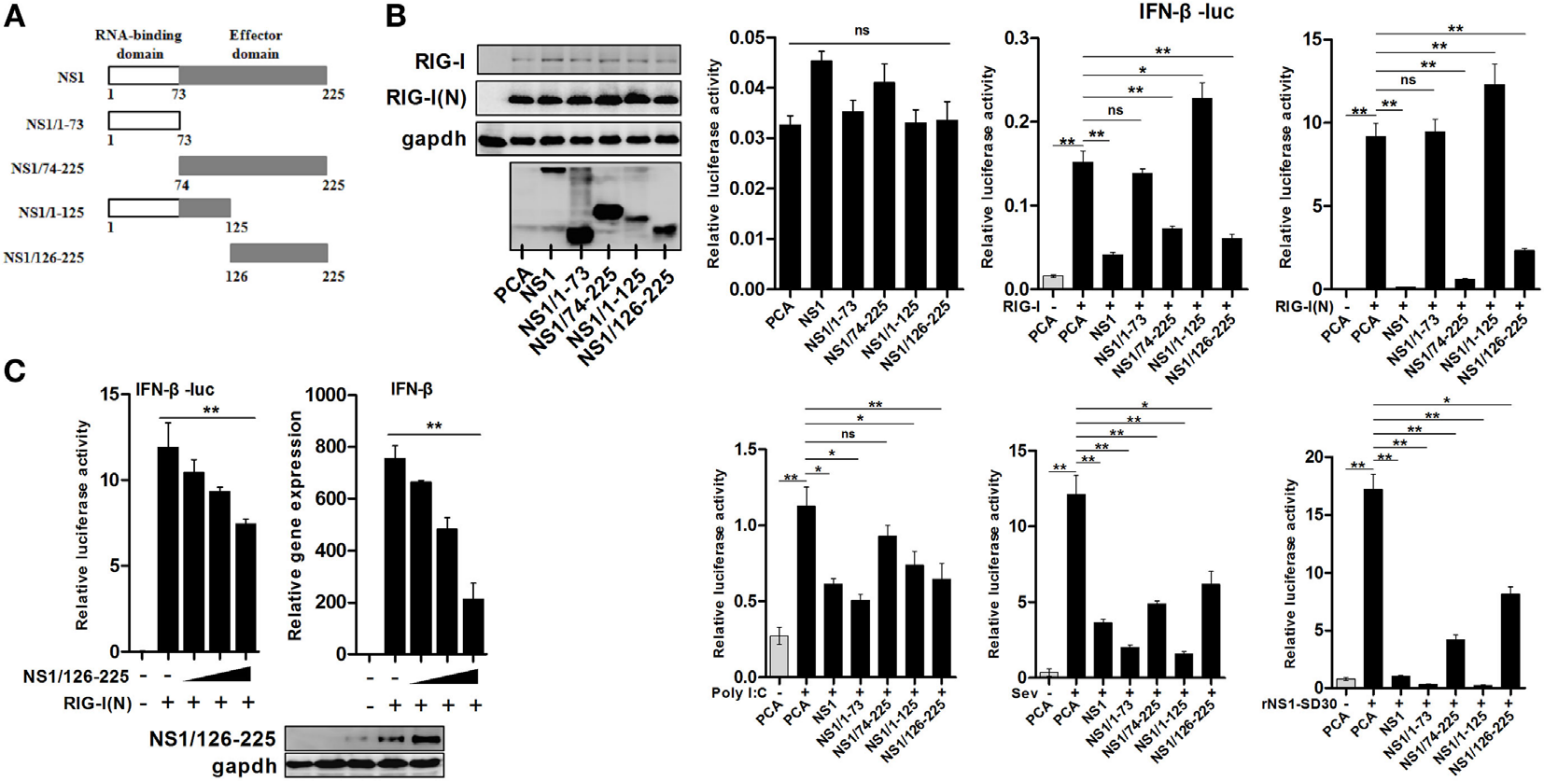
Inhibition of RIG-I(N)-induced IFN-β activation by the C-terminal effector domain (ED) of H5N1 NS1 protein. **(A)** Schematic diagram of wtNS1 protein encoded by avian H5N1 virus with the indicated truncates of the N-terminal RNA-binding domain or the C-terminal ED. **(B)** 293T cells in 24-well plates were co-transfected with 400 ng of empty vector, NS1, NS1/1-73, NS1/74-225, NS1/1-125, or NS1-126-225 along with 100 ng of IFN-β-luc and the 5 ng of pRL-TK using Lipofectamine 2000. Twenty-four hours after transfection, cells were incubated with one of the following: 50 ng of RIG-I, RIG-I(N), an empty vector for 24 h, poly(I:C) (100 ng) for 16 h, 40 HAU/ml Sev, or 0.01 MOI rNS1-SD30 virus for 16 h, then lysed with lysis buffer. Luciferase activities were detected by the dual-luciferase assay system. **(C)** 293T cells were transfected with an empty vector or increasing amounts of plasmid encoding NS1/126-225 (50, 100, 200 ng) along with RIG-I(N), IFN-β-luc, and pRL-TK. Twenty-four hours after transfection, luciferase activity and transcription of IFN-β were measured. β-actin was used as an internal control for the qRT-PCR experiments. Western blot analysis of lysates was performed. All experiments were repeated at least three times. Data shown are mean ± SD from one representative experiment. Statistical significance was analyzed with a two-tailed Student’s *t*-test (*P < 0.05 or **P < 0.01).

With the aim of elucidating the mechanism by which IAV NS1 protein counteracts the host innate immune responses, we generated four truncated H5N1 NS1 proteins, NS1/1-73, NS1/74-225, NS1/1-125, and NS1/126-225 (Figure 1A). In order to investigate the function of wtNS1, and its truncated peptides, we assessed its effect on IFN-β promoter activity using a luciferase reporter assay in 293T cells. Our results showed that wtNS1, NS1/74-225, and NS1/126-225 significantly decreased the IFN-β reporter activities driven by RIG-I or RIG-I(N). Conversely, NS1/1-73 did not change the activity of IFN-β reporter, and NS1/1-125 only slightly increased the activity compared to an empty vector control (Figure 1B). In addition, we found that wtNS1 and all truncated peptides had inhibitory effects on IFN-β reporter activities induced by Sev or rNS1-SD30 virus infection or transfection of poly(I:C) (Figure 1B). This suggests that NS1 N-terminal RBD alone is sufficient to inhibit the activation of IFN-β only in the presence of dsRNA; the C-terminal ED of NS1 could inhibit the activity of IFN-β reporter in all tested conditions. Driven by RIG-I(N), NS1/126-225 caused a dose-dependent inhibition of IFN-β promoter activity and IFN-β transcription (Figure 1C). Previous studies indicated that IAV NS1 sequesters dsRNA and binds RIG-I at its RBD, subsequently inhibiting the activation of IRF3 and preventing the induction of IFN-β (11, 16). Our findings reveal that C-terminal ED of NS1 (NS1/126-225) blocked RIG-I(N)-mediated IFN-β induction in an RNA binding-independent manner.

### NS1/126-225 Blocks RIG-I(N)-Induced IRF3 Phosphorylation and IFN-β Production

**Figure 2:**
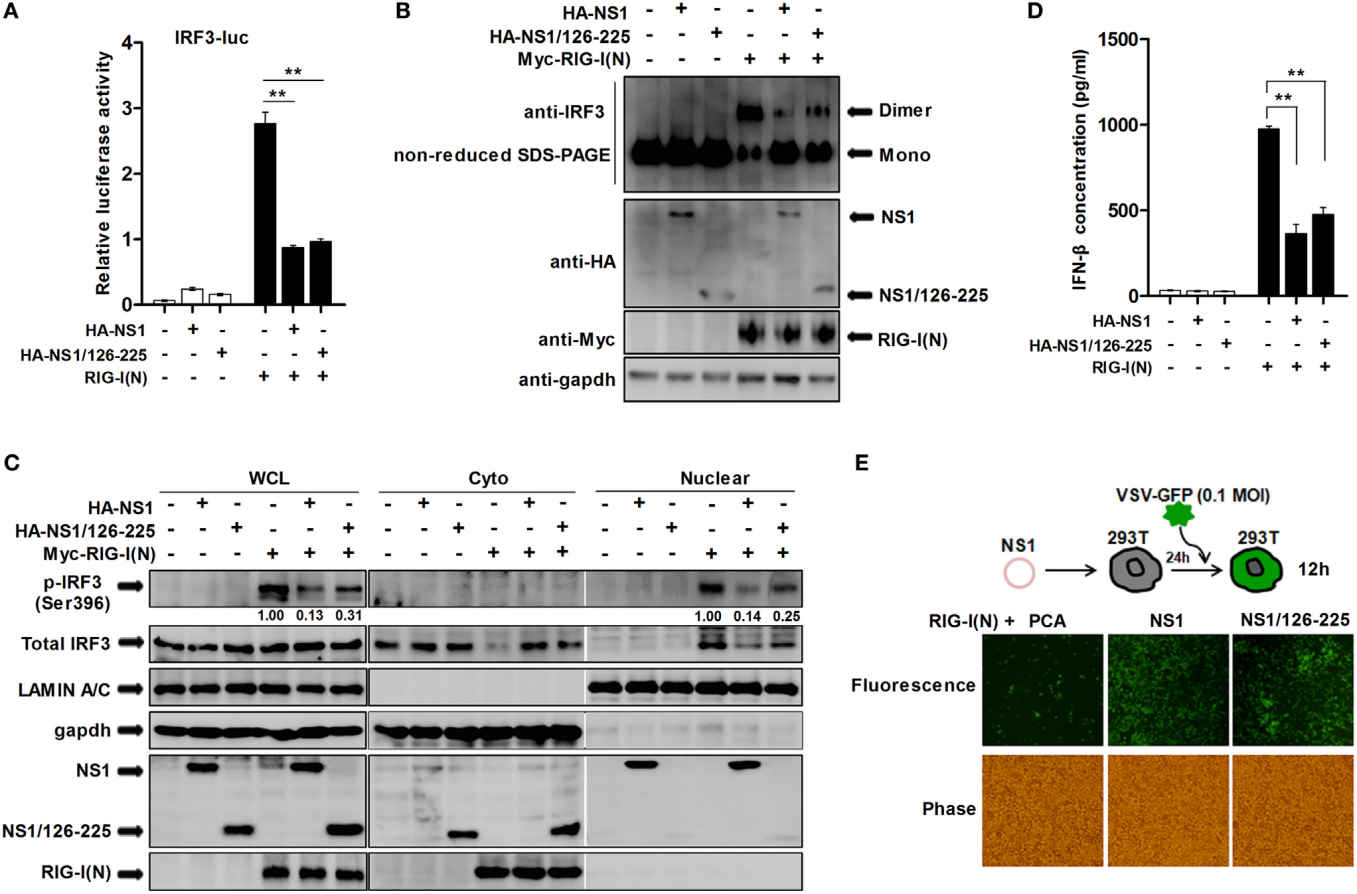
NS1/126-225 inhibits the activation of IRF3. **(A)** 293T cells were transfected with an IRF3 reporter plasmid and pRL-TK in the presence or absence of RIG-I(N). Cells were cotransfected with plasmids expressing NS1 or NS1/126-225, or an empty vector as a control. Luciferase activity within lysates was determined 24 h posttransfection. **(B,C)** 293T cells were transfected with an empty vector, NS1, or NS1/126-225 along with or without RIG-I(N) for 24 h. Lysates were then analyzed for levels of IRF3 dimers by western blotting using non-reduced SDS-PAGE **(B)**, and levels of p-IRF3(S396) and total IRF3 in the cytoplasmic and nuclear lysates **(C)**. GAPDH and Lamin A/C were used as cytoplasmic and nuclear fraction markers, respectively. **(D)** A549 cells were transfected with empty vector, NS1, or NS1/126-225 for 24 h. IFN-β production in response to RIG-I(N) was measured by ELISA. **(E)** Representative fluorescence micrographs of VSV-GFP in 293T cells transfected with an empty vector, NS1, or NS1/126-225 along with RIG-I(N) for 24 h followed by infection with VSV-GFP for 12 h. All experiments were performed at least three times with similar results. **P < 0.01 by Student’s *t*-test.

Transcription factor IRF3 is a key innate immune system component that mediates IFN-β induction. Once IRF3 is phosphorylated, it forms a dimer, translocates into the nucleus from the cytoplasm, and induces the expression of IFN-β and ISGs through specifically binding to their promoter regions (27). In order to further investigate how NS1/126-225 inhibits the signaling that mediates type I IFN production, we used an IRF3-luciferase reporter plasmid, allowing for the measurement of IRF3 activation. As shown in Figure 2A, IRF3-luciferase reporter activation by RIG-I(N) was blocked in 293T cells overexpressing NS1/126-225 or wtNS1. We next addressed whether NS1/126-225 affected the dimerization and nuclear localization of endogenous IRF3 mediated by RIG-I(N). Non-reduced SDS-PAGE and immunoblot analysis of cell lysates after RIG-I(N) transfection showed that NS1/126-225 or wtNS1 expression produced a considerable reduction in the activated dimer form of IRF3 (Figure 2B). Similarly, RIG-I(N)-induced phosphorylation of IRF3 was strongly repressed by NS1/126-225 or wtNS1 and that phosphorylated IRF3 was largely distributed in nuclear fractions (Figure 2C). ELISA assays of IFN-β in the medium of cells transfected with plasmids encoding NS1/126-225 or wtNS1 along with RIG-I(N) showed that both NS1/126-225 and wtNS1 inhibited the production of IFN-β (Figure 2D). Together, these data indicate that NS1/126-225 inhibits the expression of type I IFN induced by RIG-I(N) through blocking the phosphorylation of IRF3.

Previous studies have shown that a recombinant VSV-GFP system can be used as a strategy to screen proteins possessing IFN-antagonizing activity (28). In the present study, we employed recombinant VSV-GFP to investigate if NS1/126-225 serves as an antagonist of IFN production. When 293T cells expressed NS1/126-225, a high level of VSV-GFP replication was present, consistent with wtNS1 protein (Figure 2E), suggesting that the inhibitory effect of NS1/126-225 on IFN production is also present during actual viral infection.

### NS1/126-225 Inhibits IFN-β Signaling Pathway at the Level between MAVS and TBK1

**Figure 3:**
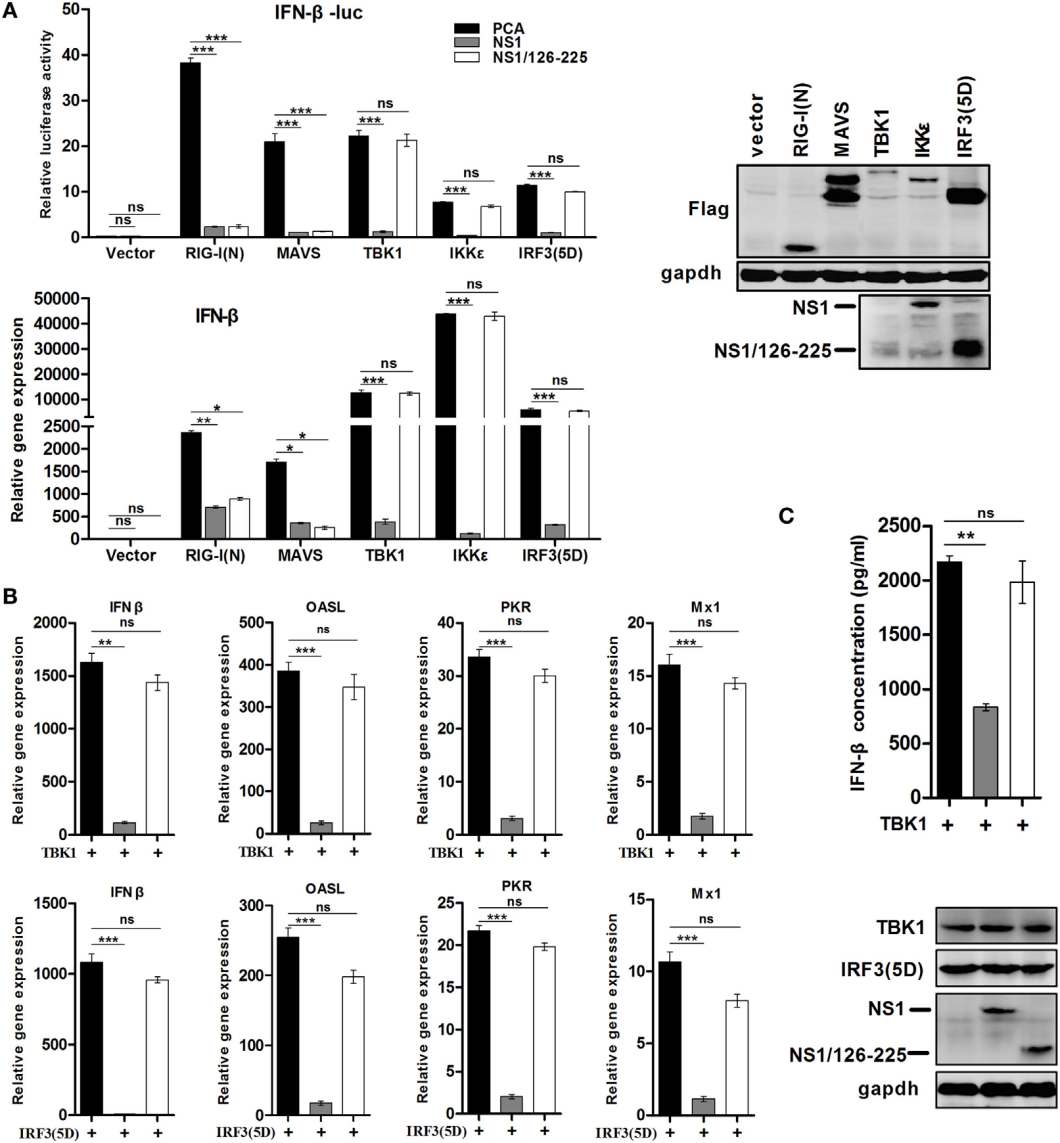
NS1/126-225 inhibits the IFN-β signaling pathway between the level of MAVS and TBK1. **(A)** 293T cells plated in 24-well plates were transfected with IFN-β-Luc, pRL-TK and empty vector, RIG-I(N), MAVS, TBK1, IKKε, or IRF3(5D) expression plasmids along with a control vector, NS1, or NS1/126-225. Luciferase activity and the transcription of IFN-β were measured at 24 h after transfection. **(B)** 293T cells plated in 12-well plates were transfected with an empty vector or a vector expressing NS1 or NS1/126-225 along with TBK1 or IRF3(5D), then IFN-β, OASL, PKR, and Mx1 mRNA levels were determined by real-time PCR. The transcript level of each gene was normalized to the expression of β-actin. **(C)** Production of IFN-β by TBK1 in the presence of NS1/126-225. A549 cells plated in 12-well plates were transfected with an empty vector, NS1, or NS1/126-225 along with TBK1. After 24 h, the amount of IFN-β in the supernatants was measured by ELISA. Western blot analysis of lysates was performed. The data are presented as mean ± SD derived from three repeat experiments. *P < 0.05 or **P < 0.01, ***P < 0.001 (Student’s *t*-test).

In order to test the effect of NS1/126-225 on various components of the RLR pathway, NS1/126-225 and expression plasmids of RIG-I signaling pathway components, including RIG-I(N), MAVS, TBK1, IKKε, and the active form of IRF3 [IRF3(5D)], were cotransfected into 293T cells. IFN-β promoter activity and transcription levels of IFN-β were then detected. Reporter assay results showed that NS1/126-225 inhibited the IFN-β activity induced by RIG-I(N) or MAVS, whereas NS1/126-225 could not suppress IFN-β promoter activation driven by TBK1, IKKε, or IRF3(5D) (Figure 3A). By contrast, wtNS1 significantly decreased the RLR adaptor-mediated IFN-β promoter activity. As expected, NS1/126-225 exhibited a decrease in the IFN-β mRNA level induced by RIG-I(N) or MAVS (Figure 3A). In addition, the secretion of IFN-β and the transcription level of ISGs triggered by TBK1 or IRF3(5D) in the presence of NS1/126-225 were tested. NS1/126-225 induced nearly a complete loss of the inhibition of IFN-β and ISGs, including OASL, PKR, and Mx1, whereas wtNS1 strongly blocked the production of IFN-β and ISGs mRNA expression (Figures 3B,C). Together, these data indicate that NS1/126-225 significantly inhibits the cellular antiviral response at the level between MAVS and TBK1.

### NS1/126-225 or wtNS1 Interacts with TRAF3

**Figure 4:**
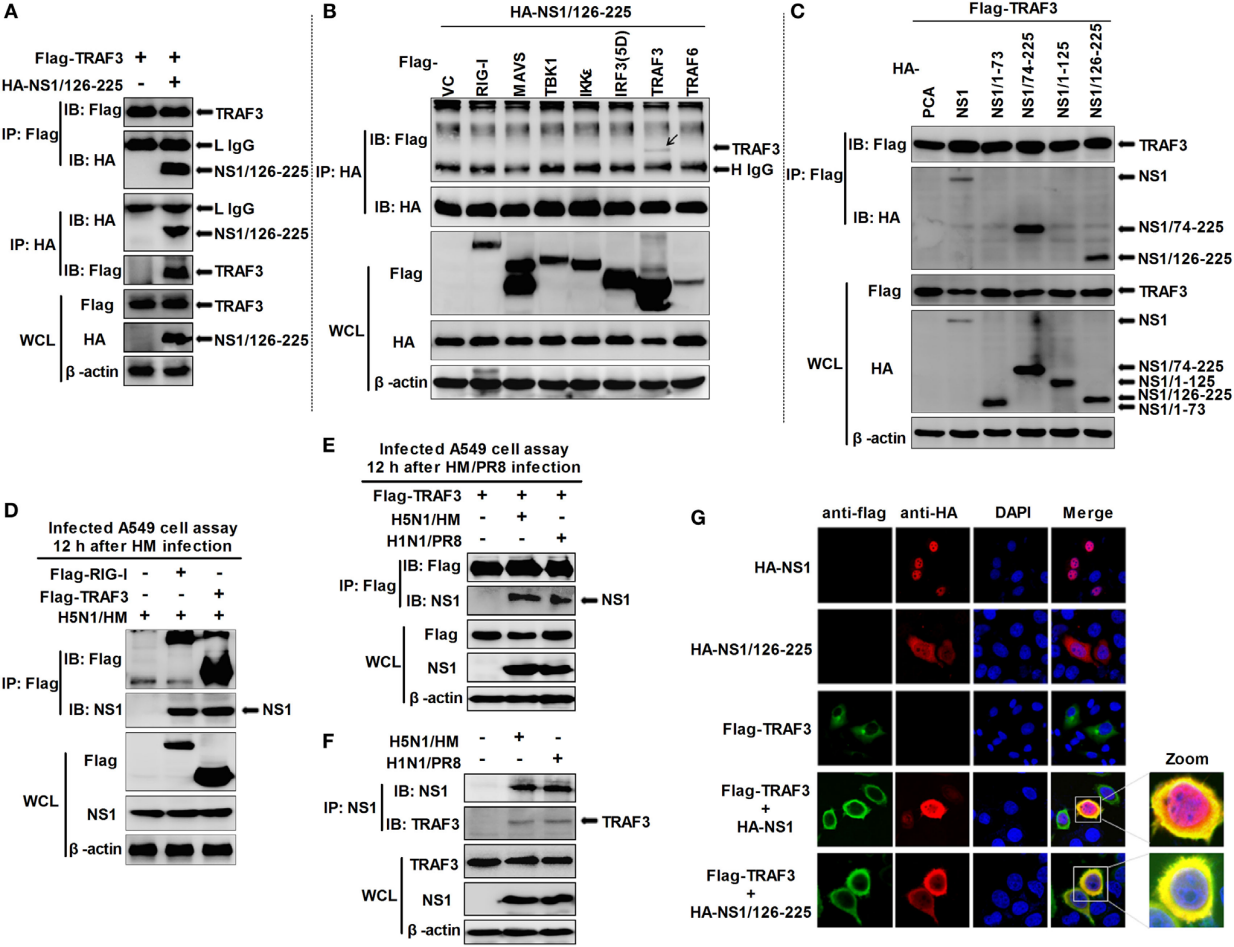
Identification of NS1/126-225 or wtNS1 that bind TRAF3. **(A)** 293T cells seeded into 60-mm dishes and transfected with a plasmid expressing HA-NS1/126-225 along with Flag-TRAF3 for 36 h. **(B)** 293T cells were transfected with HA-NS1/126-225 and different components of the RLR signaling pathway for 36 h. **(C)** 293T cells were transfected with NS1 and their truncations, along with Flag-TRAF3 for 36 h. Cell lysates were harvested and subjected to immunoprecipitation using an anti-Flag or anti-HA antibody, and immunoblot analyses were performed with the indicated antibodies. **(D)** A549 cells were transfected for 24 h with plasmids Flag-TRAF3, Flag-RIG-I, or an empty vector. Cells were infected with two MOI of HM virus for 12 h. **(E)** A549 cells were transfected for 24 h with Flag-TRAF3. Then cells were infected with 2 MOI of HM or PR8 virus for 12 h. Cell extracts were subject to immunoprecipitation with anti-Flag antibodies. Aliquots of the eluates were immunoblotted using the anti-NS1 antibody. **(F)** A549 cells were infected with either HM, PR8 at two MOI, or mock infected for 12 h, then whole-cell lysates were subjected to immunoprecipitation using an anti-NS1 antibody and analyzed by western blotting. Expression of the transfected proteins was determined by western blotting using the indicated antibodies (bottom). **(G)** NS1/126-225 or wtNS1 colocalized with TRAF3 in the cytoplasm. Following transfection with HA-NS1/126-225, HA-NS1, and Flag-TRAF3, HeLa cells were fixed with 4% paraformaldehyde and stained with anti-HA or anti-Flag antibodies. Secondary antibodies conjugated to Cy3 or FITC were used to visualize the stained NS1/126-225, NS1, and TRAF3 proteins, respectively. Diamidino-2-phenylindole shows the nuclei of cells. Fluorescence was examined with a Zeiss LSM 510 Meta confocal microscope.

In the RLR-mediated signaling pathway, TRAF3 serves as a critical link between the adaptor MAVS and downstream regulatory kinases that are essential for IRF3 activation (29, 30). Thus, we hypothesized that TRAF3 is the target of NS1/126-225. Co-IP experiments revealed that NS1/126-225 interacted selectively with TRAF3 but not other components (Figures 4A,B). In another experiment, wtNS1 and NS1/74-225 also interacted most potently with TRAF3 (Figure 4C). This association was confirmed under physiological conditions in an experiment that detected this interaction by overexpression of Flag-TRAF3 in infected A549 cells, where RIG-I served as a positive control (Figure 4D). Furthermore, PR8 NS1 also interacted with TRAF3 in infected A549 cells (Figure 4E). To address whether the wtNS1 protein physically interacts with endogenous TRAF3, we performed endogenous IP assays on H5N1/HM or PR8-infected cell lysates. Our results showed that TRAF3 could be co-precipitated by the NS1 antibody (Figure 4F). In addition, the NS1 proteins of the strain A/Shanghai/02/2013(H7N9) and other avian H9N2 strain did not bind TRAF3 (Figure S1 in Supplementary Material). These results demonstrate that NS1/126-225 or wtNS1 interacts with TRAF3 in a strain-specific manner.

Based on the findings that NS1/126-225 or wtNS1 interacted with TRAF3, we next asked whether the two molecules co-localize in cells. Confocal microscopy revealed the co-staining of NS1/126-225 or wtNS1 and TRAF3 in cells, suggesting the co-localization of the two proteins (Figure 4G). Normally, expression of wtNS1 in HeLa cells resulted in a nuclear localization with minor cytoplasmic staining. TRAF3 overexpression led to a marked increase in the cytoplasmic localization of wtNS1 or NS1/126-225. These results indicate that NS1/126-225 or wtNS1 colocalize with TRAF3 in cells and TRAF3 overexpression led to a marked increase of NS1/126-225 or wtNS1 cytoplasmic localization.

### TRAF3 Is Essential for NS1/126-225 to Downregulate IFN-β

**Figure 5:**
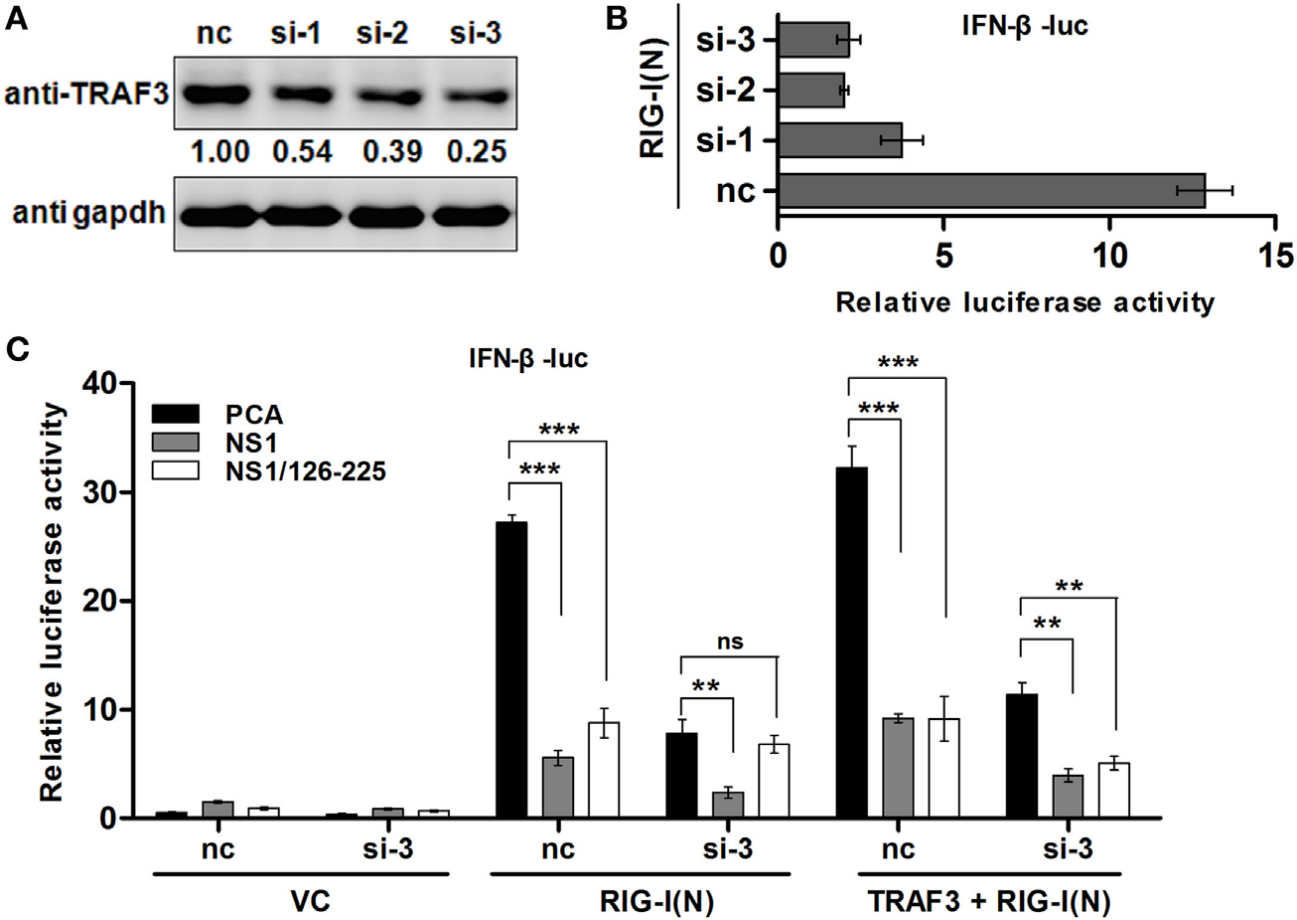
TRAF3 is necessary for NS1/126-225 to downregulate IFN-β. **(A)** 293T cells were transfected with three TRAF3 siRNAs (60 pmol) for 36 h. Cell lysates were detected using an anti-TRAF3 antibody. **(B)** Three TRAF3 siRNAs or a negative control siRNA was transfected for 24 h with IFN-β-luc and pRL-TK along with RIG-I(N) in 293T cells. Luciferase reporter assay was performed to examine the activation of IFN-β in cells. **(C)** 293T cells were transfected with negative control siRNA or TRAF3 siRNA (si-3) for 24 h, then transfected with IFN-β-luc and pRL-TK, along with the vector, NS1/126-225, or NS1 together with or without RIG-I(N) or RIG-I(N) and TRAF3 for 24 h. The promoter activities were detected by the dual-luciferase assay system. All luciferase assays were repeated at least three times, and the data shown are mean ± SD from one representative experiment. Significance was analyzed with a two-tailed Student’s *t*-test (*P < 0.05 or **P < 0.01, ***P < 0.001).

The above data showed that NS1/126-225 blocks IFN-β induction and interacts with TRAF3. Hence, we used TRAF3 siRNA to determine whether TRAF3 is essential for the regulatory function of NS1/126-225 in 293T cells. Knockdown of TRAF3 by siRNA diminished IFN-β promoter activity triggered by RIG-I(N) (Figures 5A,B). In cells with silenced TRAF3, after transfection of RIG-I(N), the induction of IFN-β was greatly reduced and the inhibitory effect of NS1/126-225 was markedly attenuated. Nevertheless, this inhibitory effect was rescued successfully with a TRAF3 expression plasmid (Figure 5C). These data suggest that TRAF3 is necessary for NS1/126-225 to decrease IFN-β activity.

### NS1/126-225 Suppresses TRAF3 K63-Linked Ubiquitination

**Figure 6:**
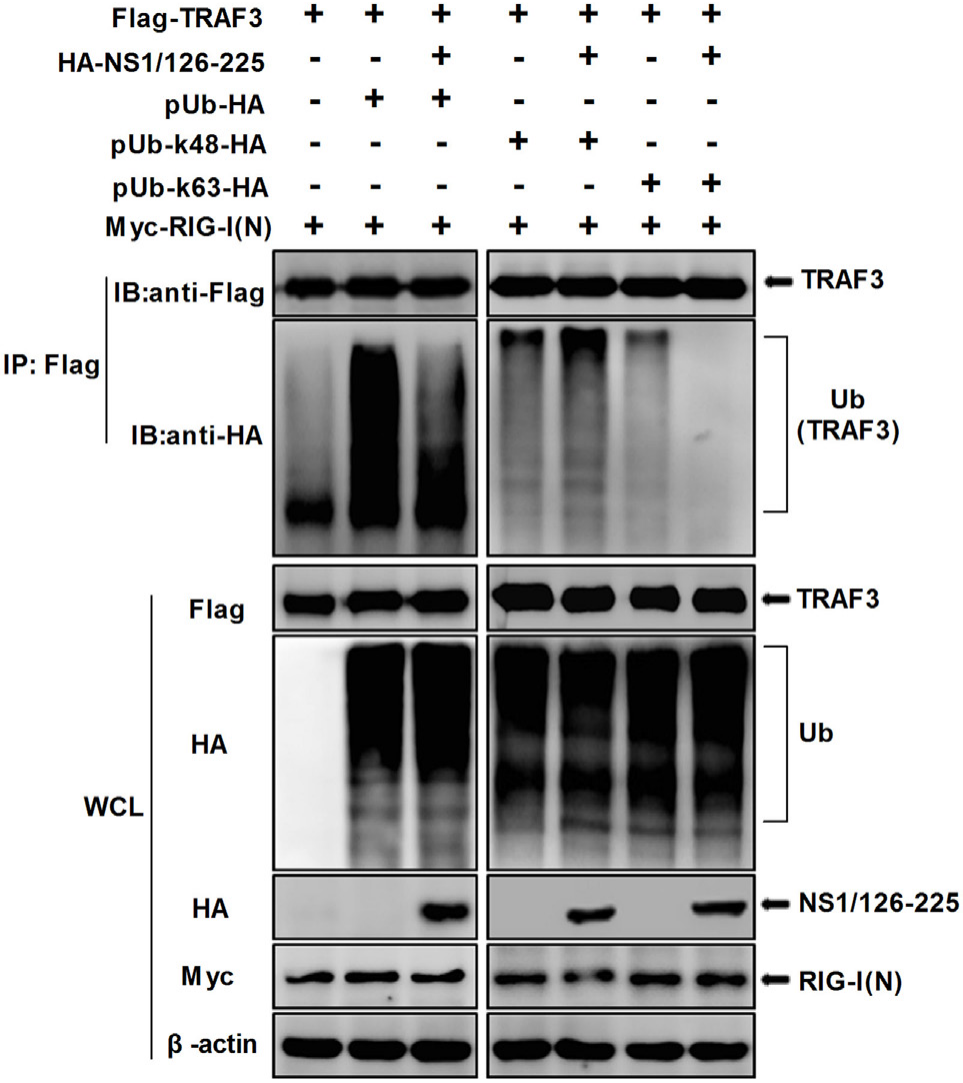
NS1/126-225 inhibits TRAF3 K63-linked ubiquitination. 293T cells were transfected with Flag-TRAF3, RIG-I(N), pUb-HA, together with or without HA-NS1/126-225, or with ubiquitin mutants pUb-K48-HA and pUb-K63-HA. Thirty-six hours later, cells were subjected to anti-Flag immunoprecipitation and immunoblotting with anti-HA to monitor the ubiquitination of TRAF3.

It is well-known that TRAF3 is modified with a polyubiquitin chain to provide a scaffold for complex formation. Thus, in order to study the effect of NS1/126-225 on TRAF3 ubiquitination, Co-IP experiments were conducted. Flag-TRAF3, pUb-HA, and NS1/126-225 along with RIG-I(N) were cotransfected into 293T cells and the ubiquitination level of TRAF3 was monitored. Compared to the control, NS1/126-225 reduced the RIG-I(N)-induced ubiquitination of TRAF3 (Figure 6, left). To explore the type of TRAF3 ubiquitin chains, Flag-TRAF3 was transfected into 293T cells with ubiquitin mutants, including the pUb-K48-HA or pUb-K63-HA expression plasmid. The results showed that NS1/126-225 could decrease the K63-linked ubiquitination of TRAF3 but not the K48-linked ubiquitination of TRAF3 (Figure 6, right). In summary, these results demonstrate that NS1/126-225 suppresses the K63-linked ubiquitination of TRAF3 that is important for the recruitment of the TBK1-IKKε kinase complex.

### NS1/126-225 Interacts with the TRAF Domain of TRAF3 and Impairs the Formation of MAVS–TRAF3 Complex

**Figure 7:**
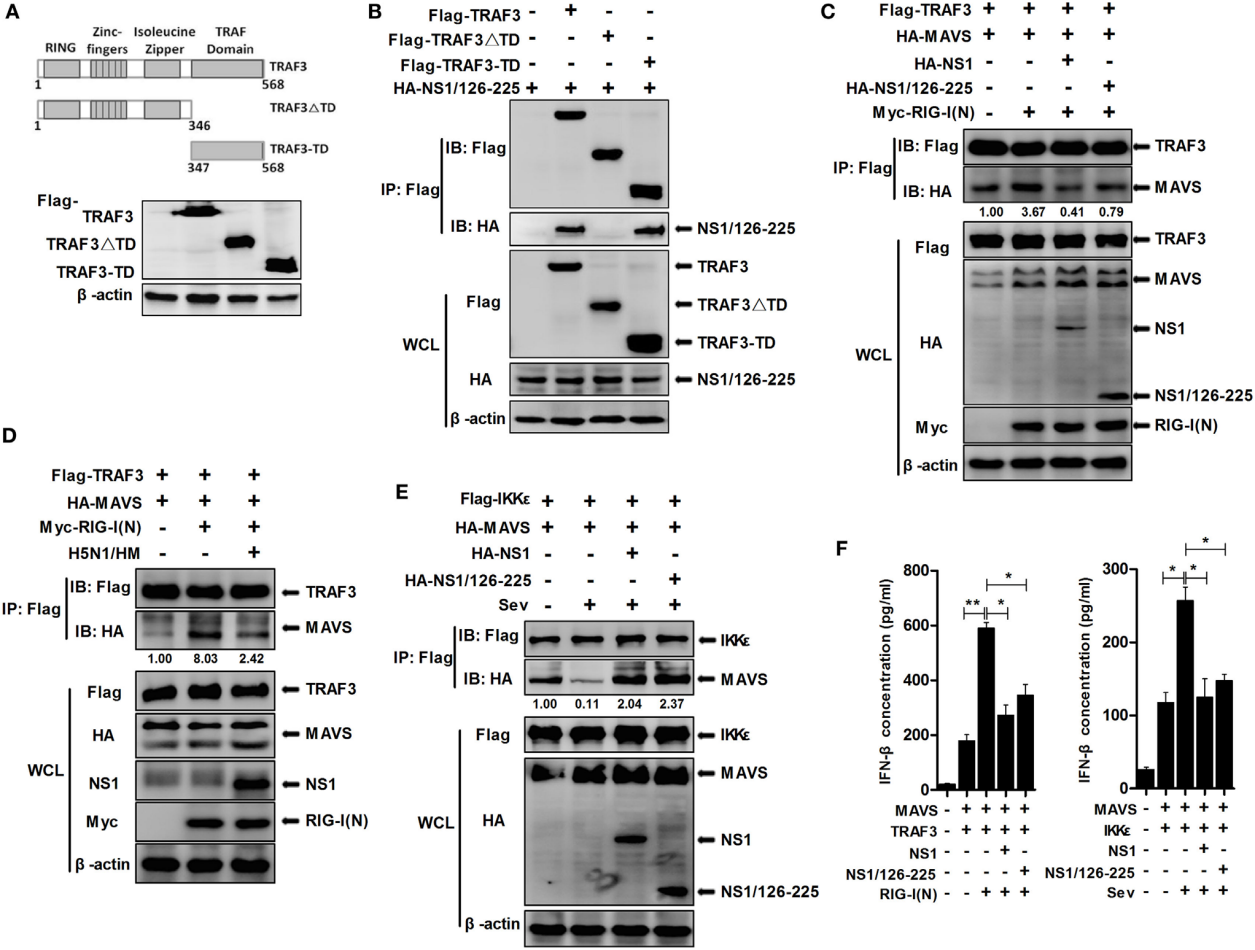
NS1/126-225 disrupts the formation of MAVS–TRAF3 complex. **(A)** Schematic diagram of the TRAF3 protein and the following deletion mutants: Flag-TRAF3-ΔTD (AA1 to 346) and Flag-TRAF3-TD (AA347 to 568). **(B)** Co-IP and western blotting of 293T cells transfected with HA-tagged NS1/126-225 along with a vector expressing the indicated Flag-tagged TRAF3 truncations or full-length TRAF3. An empty vector was used as a negative control. **(C)** 293T cells were transfected with Flag-TRAF3 and HA-MAVS together with either HA-NS1/126-225 or HA-NS1 and Myc-RIG-I(N) for 36 h. **(D)** 293T cells were transfected with Flag-TRAF3 and HA-MAVS together with or without Myc-RIG-I(N) for 24 h, then infected with 0.1 MOI HM virus for 12 h. Cell lysates were harvested, then subjected to immunoprecipitation using an anti-Flag antibody, and analyzed by western blotting. **(E)** 293T cells were transfected with HA-NS1/126-225 as well as Flag-IKKε and HA-MAVS for 24 h. Cell lysates were collected 12 h post-infection with Sev. Co-IP was performed with anti-Flag, and the precipitates were probed with anti-Flag and anti-HA antibodies. **(F)** A549 cells in 12-well plates were transfected with NS1/126-225, NS1 or an empty vector along with MAVS and TRAF3, together with or without RIG-I(N) for 36 h; NS1/126-225 or NS1 cotransfected with MAVS and IKKε for 24 h, then cells were infected with Sev or mock infected for 12 h. IFN-β concentrations in the supernatants were measured by ELISA. All experiments were performed at least three times. Statistical significance was analyzed with a two-tailed student’s *t*-test (*P < 0.05 or **P < 0.01).

TNF receptor-associated factor 3 consists of four structural and functional domains; a RING domain, a Zinc-fingers domain, an Isoleucine Zipper domain, and a TRAF domain. To map the TRAF3 domains involved in NS1/126-225 binding, we generated the following TRAF3 deletion mutants: Flag-TRAF3-ΔTD, expressing AA1 to 346 and containing the RING domain, the Zinc-fingers domain, the Isoleucine Zipper domain, and Flag-TRAF3-TD, expressing AA347 to 568 and containing the TRAF domain (Figure 7A). Co-IP assays showed that the TRAF domain of TRAF3 was found to be the crucial region responsible for the association of TRAF3 with NS1/126-225 (Figure 7B) as well as with wtNS1 (data not shown).

Previously, it has been reported that the TIM domain of MAVS interacts with amino acid residues Y440 and Q442 within the TRAF domain of TRAF3 (31). To examine whether NS1/126-225 affected IFN signaling at the level of MAVS–TRAF3 interaction, MAVS–TRAF3 association was determined in the presence of NS1/126-225. Expression of MAVS led to an interaction with TRAF3 and was increased by RIG-I(N) transfection. However, NS1/126-225 and wtNS1 markedly disrupted this interaction by 4.6- and 8.5-fold, respectively (Figure 7C). In H5N1/HM infection of A549 cells, the MAVS–TRAF3 complex was decreased by 3.3-fold compared to the control (Figure 7D).

As reported previously, IKKε is recruited to the C-terminal region of MAVS following Sev or VSV infection, mediated by Lys63-linked polyubiquitination of MAVS at Lys500, resulting in the inhibition of downstream IFN signaling (31, 32). Therefore, it was necessary to test whether wtNS1 or NS1/126-225 affect this process to accomplish its negative regulatory role in IFN-β production after Sev infection. The interaction of MAVS and IKKε was readily detected by Co-IP, while Sev infection resulted in a significant decrease in the MAVS–IKKε interaction. Interestingly, the interaction of MAVS and IKKε was remarkably increased when cotransfected with NS1/126-225 or wtNS1 (Figure 7E), indicating that NS1/126-225 or NS1 promotes the recruitment of IKKε to MAVS. Furthermore, we measured the secretion of IFN-β in cell supernatants. Results showed that NS1/126-225 or NS1 inhibited the production of IFN-β mediated by MAVS–TRAF3 or MAVS–IKKε (Figure 7F), implying that NS1/126-225 functions in downregulating IFN expression. Taken together, these results indicate that the association of the MAVS–TRAF3 complex is disrupted by NS1/126-225, which, in turn, blocks IFN-β production.

### NS1/126-225 Increases Virus Replication in AIV-Infected A549 Cells

**Figure 8:**
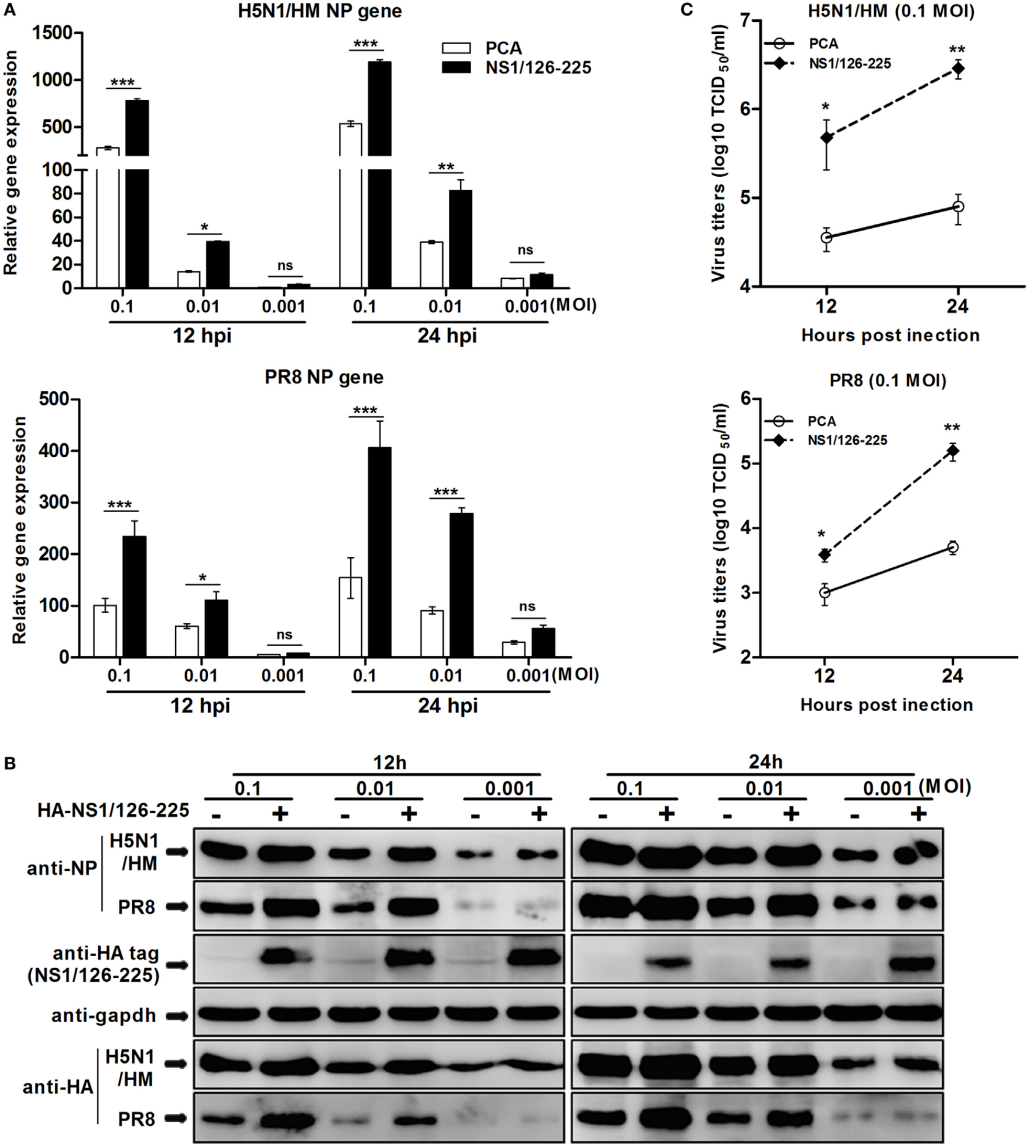
NS1/126-225 increases virus replication in IAV-infected A549 cells. **(A)** A549 cells were transfected with HA-NS1/126-225 or an empty vector and infected with HM or PR8 virus at the indicated MOIs (0.001, 0.01, and 0.1) at 24 h posttransfection. Total RNA was extracted to examine the mRNA levels of NP transcripts by qPCR at the indicated time points (12 and 24 h). β-actin was used as an internal control for normalization, and the relative expression is represented as fold induction relative to the expression level in control cells (set to 1). **(B)** Western blotting was performed to detect protein levels of NP and HA. **(C)** Culture supernatants from cells infected with HM or PR8 (0.1 MOI) were collected, and viral titers was measured by TCID_50_. Results shown are representative of three independent experiments. The data are presented as the means ± SD derived from three repeat experiments. *P < 0.05 or **P < 0.01, ***P < 0.001 (Student’s *t*-test).

To determine whether the replication of IAV is enhanced by the NS1/126-225 protein, A549 cells were transfected with NS1/126-225, or an empty vector, then infected with different titers of H5N1/HM or PR8 virus. Upon infection with H5N1/HM or PR8 virus, NS1/126-225 could facilitate transcription of NP of both viruses (Figure 8A), resulting in an increase in NP and HA proteins observed in the NS1/126-225 group (Figure 8B). This was confirmed by the titers of H5N1/HM or PR8 virus, which significantly increased by 40- and 31-fold, respectively, in NS1/126-225 overexpressing cells compared with control cells (Figure 8C). These results demonstrate that NS1/126-225 enhances the capacity of IAV to replicate in cells.

## Discussion

PRRs of host cells recognize a PAMP and subsequently initiate a series of signaling cascades. The final step is activation of IRF3 and NF-κB, thus inducing the transcription of IFN-β (33). Given the cascade of responses triggered by the host in response to infection, influenza viruses adapted different strategies to escape the IFN response. This survival tactic has proven successful in order for virus proliferation and infection. Both PB2 and PB1-F2 limit IFN production by associating with MAVS (10, 34, 35). Other structural proteins, such as PB1, PA, NP, and even the genomic RNA itself, also contribute to impairing RIG-I-mediated antiviral responses (36). Moreover, HA (HA1) was recently shown to drive the degradation of the IFN receptor chain IFNAR1, thereby suppressing IFN-triggered JAK/STAT signaling (37). The most effective weapon influenza A viruses have at their disposal is NS1 protein.

**Figure 9:**
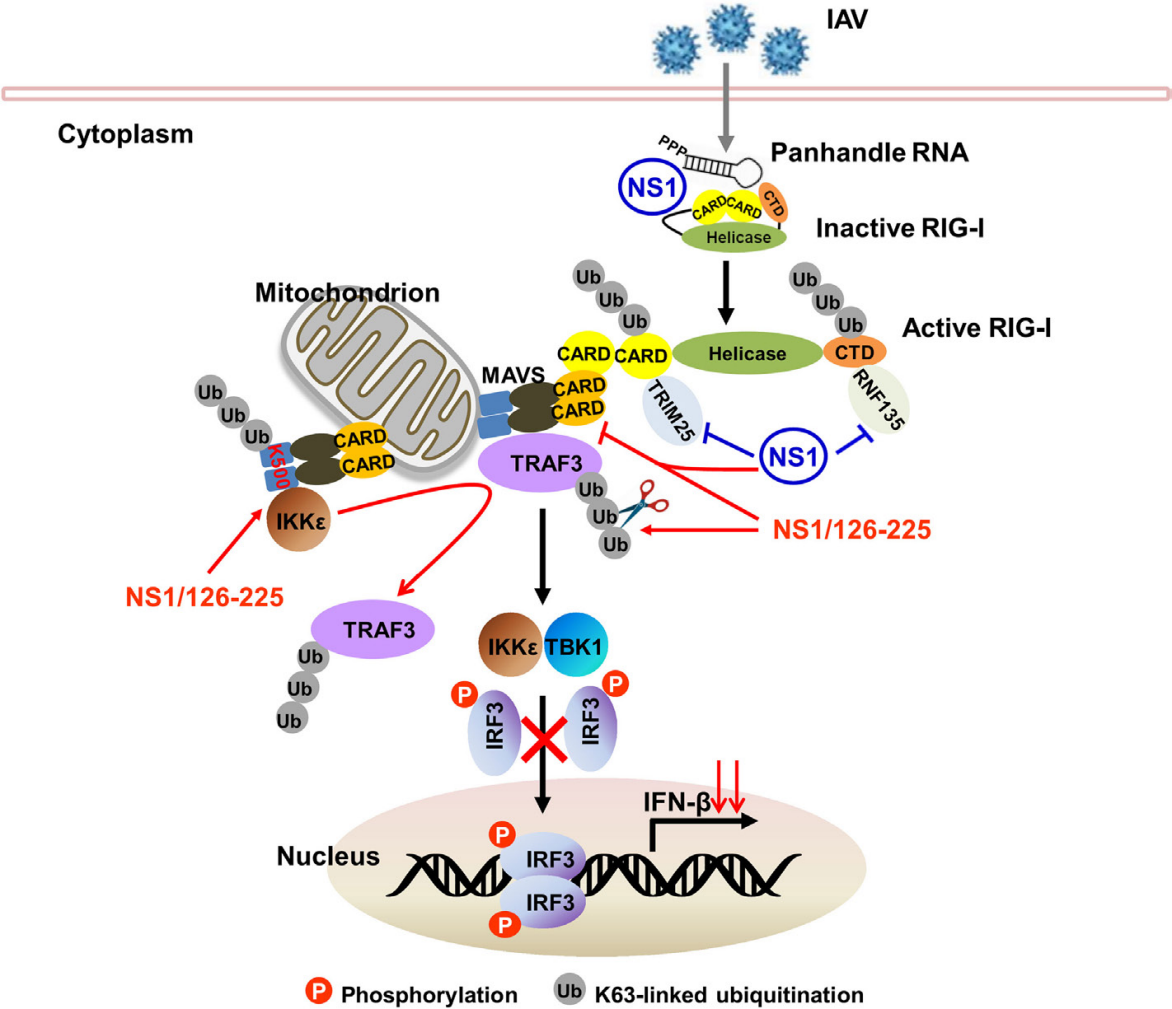
Model of TRAF3-dependent regulation of RIG-I signaling pathway by NS1/126-225. Following IAV infection, RIG-I recognizes and binds 5′-triphosphate dsRNA to initiate host antiviral response. The NS1 protein of IAV inhibits host IFN responses either by sequestering viral dsRNA or by binding to RIG-I and TRIM25 or RIPLET proteins required for RIG-I activation. In addition, the NS1 C-terminal effector domain (AA126 to 225, NS1/126-225) associates with TRAF3 to remove the Lys63-polyubiquitin chains on TRAF3 and to disrupt the MAVS–TRAF3 complex. NS1/126-225 also increases the recruitment of IKKε to MAVS. This releases TRAF3 from the mitochondria, further decreasing the level of K63-linked ubiquitination of TRAF3 and impairing IRF3 phosphorylation. This results in a decreased level of IFN-β transcription.

The NS1 protein acts as an antiviral antagonist protein capable of limiting IFN production. RIG-I recognizes and binds dsRNA structures with 5′-triphosphates upon infection to initiate the host antiviral response. During the course of viral infection, the NS1 protein of IAV inhibits host IFN responses either by sequestering viral dsRNA or by binding to RIG-I and TRIM25 or RIPLET proteins required for RIG-I activation and IFN signaling pathways (11, 16, 18, 19). In this study, we found that the NS1 C-terminal ED (AA126 to 225) of H5N1 virus inhibits the activation of IFN-β pathway. To achieve a negative regulatory function in the cellular antiviral response, NS1/126-225 associates with TRAF3 to remove the Lys63-polyubiquitin chains on TRAF3 and to disrupt the MAVS–TRAF3 complex. NS1/126-225 also increases the recruitment of IKKε to MAVS, releasing TRAF3 from the mitochondria. This further decreases the level of K63-linked ubiquitination of TRAF3, impairing IRF3 phosphorylation and reducing the production of IFN-β (Figure 9).

Interestingly, our study has shown that the ED of NS1 protein possesses the ability to suppress IFN response in the absence of RNA. Typically, the RBD of NS1 mediates the inhibition of IFN synthesis, and the ED of NS1 induces the inhibition of gene expression, together with its known interactors (11). In this study, we have found that the NS1 C-terminal ED (NS1/74-225 and NS1/126-225), but not the RBD (NS1/1-73 and NS1/1-125) block IFN-β reporter activity induced by RIG-I(N). Expression of NS1/126-225 resulted in the inhibition of IRF3 activation, indicating that the NS1 protein blocks IFN-β activation through an RNA-independent manner. It was demonstrated in previous studies that influenza A viruses TX/98 and A/Viet Nam/1203/04 expressing C-terminally truncated NS1 proteins of 73, 99, or 126 amino acids were attenuated. The resultant reduced growth correlated with a high level of IFN-α/β induced by these mutant viruses (28, 38). In addition, in both influenza B and C viruses, the C-terminal domains of the NS1 proteins were found to possess IFN antagonist activity (39, 40). More importantly, the N terminus-truncated NS1 proteins encoded by PR8, which was translated from internal AUGs at positions 235 and 241 of the NS1 open reading frame, were found to inhibit the activation of IRF3 and IFN-β transcription (41). This observation is supported by our findings that the influenza A virus NS1 ED plays an important role in the ability of the NS1 protein to inhibit IFN-β pathway.

To elucidate the mechanism of how the NS1 ED inhibits IFN-β activation, we speculated that the NS1 ED contacts its counterparts in RIG-I signaling leading to inhibition. For this purpose, we examined a step within the signaling pathway that NS1/126-225 targets and found that NS1/126-225 acted downstream of MAVS and upstream of TBK1. The Co-IP assays showed unexpectedly that NS1/126-225 binds to TRAF3, which interacts with MAVS forming a platform for RNA virus signaling. We also tested the binding of TRAF3 to the full-length NS1 protein and found that this interaction exists, and co-localized in the cytoplasm. The NS1/126-225 protein mainly localized in the cytoplasm. TRAF3 expression led to a marked increase in the NS1/126-225 cytoplasmic localization, suggesting that NS1/126-225 inhibits the activation of the IFN-β pathway. Although all types of influenza virus NS1 proteins interact with TRIM25, only part of NS1 prevents IRF3 activation, indicating that TRIM25 is not required for the inhibition of IRF3 activation. In this study, we did not observe the interaction between NS1/126-225 and RIG-I, consistent with the results described in a previous publication (41). Consequently, RIG-I seems to be non-essential for the optimal inhibition of IFN production in IAV-infected cells. However, our study demonstrated that the influenza A virus NS1 ED targets TRAF3, subsequently inhibits IFN production, implying that TRAF3 is a key factor involved for IAV to escape host innate immune responses.

The MAVS–TRAF3 complex is a focal point of RLR-directed signaling response (42, 43). TRAF3 localizes to the endoplasmic reticulum (ER) and needs to be recruited to mitochondrial MAVS in order to activate TBK1 complexes (44). Many viral proteins, accessory and non-structural proteins in particular, hijack TRAF3 or the TRAF3 complex to mediate immune evasion. SARS coronavirus M protein or Open Reading Frame-9b prevents the formation of TRAF3–TANK–TBK1/IKKε complex or MAVS–TRAF3/TRAF6 signalosome to evade host innate immunity (45, 46). SARS-CoV papain-like protease interacts with and disrupts STING–TRAF3–TBK1 complex, it also inhibits the TLR7-mediated innate immunity through removing Lys63-linked ubiquitin chains of TRAF3 and TRAF6 (47, 48). Herpes simplex virus 1 ubiquitin-specific protease UL36 deubiquitinates TRAF3 then counteracts the IFN-β pathway (49). Over the past 10 years, there have been major advances in understanding how influenza A viruses successfully escape the surveillance of the immune system. The current report furthers this research revealing the surprising finding that NS1/126-225 acts by targeting TRAF3; specifically, NS1/126-225 targets the TRAF domain of TRAF3. TRAF3 links the upstream IFN signaling responses of MAVS to TBK1 relying on the TRAF domain. This report also shows that a specific interaction between TRAF3 and MAVS was observed when TRAF3 and MAVS were co-expressed in 293T cells. However, the interaction between TRAF3 and MAVS was disrupted in the presence of NS1/126-225. Interestingly, the interaction between MAVS and IKKε was markedly increased in NS1/126-225-expressing cells. It has been previously demonstrated that, after Sev infection, K63-linked polyubiquitination at Lys500 of MAVS recruits IKKε to the mitochondria, functionally causes release of TRAF3 from MAVS initiating the signal to shutdown the IFN response (31). The MAVS–IKKε complex was enhanced when NS1/126-225 was present, indicating that NS1/126-225 can utilize this process to shut down further activation of IFN pathway. Taken together, these data indicate that NS1/126-225 impedes the interactions between components of MAVS–TRAF3 complex, preventing the phosphorylation of IRF3, where it would activate the IFN-β response.

Ubiquitination has emerged as a key posttranslational modification that controls induction and shutdown of the interferon response. TRAF3, serving as a crucial functional link, is modified with a polyubiquitin chain providing a scaffold for complex formation, and, not surprisingly, many viruses encode proteins that inhibit ubiquitination processes to overcome host innate responses. Previous studies showed that nairoviruses and arteriviruses encode for ovarian tumor domain-containing proteases that hydrolyze ubiquitin chains from host proteins (50, 51). In this report, we have shown that NS1/126-225 suppresses the K63-linked ubiquitination of TRAF3. It is likely that NS1/126-225 works through recruiting a deubiquitinase to cleave the TRAF3 ubiquitin chain since it has been shown that NS1/126-225 does not belong to any known deubiquitinase family. For example, DUBA, a member of the Otubain (OTUB) family, has been shown to deubiquitinate TRAF3 and negatively regulate TLR3- and RIG-I/MDA5-mediated IFN induction (52). It was also shown that two OTUB deubiquitinating enzyme family members, OTUB1 and OTUB2, can deubiquitinate TRAF3 and TRAF6, leading to the inhibition of virus-induced IFN-β expression and cellular antiviral responses (53). Therefore, whether these proteins, or other DUB proteins, are involved in this regulation, and the detailed regulatory mechanism of TRAF3 activity triggered by NS1/126-225 remains to be discovered.

Interestingly, strain-specific targeting of TRAF3 was demonstrated by specific interaction of NS1 proteins encoded by PR8 or avian H5N1 but not novel H7N9 or avian H9N2 viruses. This difference may be associated with strain-specific sequence variations. The NS1 protein most often occurs as a 230 residue peptide, including NS1 of seasonal H1N1 virus and avian H5N1 virus (80–84 residues have been deleted since 2000), which were used in this study. However, premature stop codons or, alternatively, suppression of the genuine stop codon (codon 231) resulted in length variations at NS1’s C-terminus. Abdelwhab et al. analyzed NS1 protein sequences of all AIV subtypes in birds from 1902 to 2015 to study the prevalence and distribution of carboxyl terminal end truncation (ΔCTE). They found that NS217 proteins lacking amino acids 218-230 were the most prevalent form (88%). This truncation is prevalent in LPAIV of non-H5/H7 subtypes; particularly H9N2, H10, and H6 viruses that are known to be widespread and mostly (semi)endemic in land-based poultry (54). Similar truncations have also been observed in swine influenza viruses, which harbor a C-terminally truncated NS1 and have also been found in human H1N1 viruses that have been in public circulation since the 2009 pandemic (55). Hence, whether the interaction of NS1 and TRAF3 are associated with the ΔCTE requires further investigation.

In summary, the present study demonstrated that TRAF3 is a target of the C-terminal ED (AA126 to 225) of H5N1 NS1 protein, revealing a novel function of the NS1 protein in modulating host innate immunity and possibly facilitating IAV infection. The physiological significance of the NS1 ED in IAV replication and its pathological role in flu diseases warrant further investigation to probe the potential value of this molecule as a therapeutic and/or disease prevention target.

## Author Contributions

MJ and WQ conceived and designed the experiments. WQ, XW, KG, YL, XL, ZZ, and HZ performed the experiments and analyzed data. WQ and MJ wrote the manuscript. All authors reviewed, revised, and approved the final manuscript.

## Conflict of Interest Statement

The authors declare that the research was conducted in the absence of any commercial or financial relationships that could be construed as a potential conflict of interest. The reviewer, BN, and handling editor declared their shared affiliation, and the handling editor states that the process nevertheless met the standards of a fair and objective review.
