# Supplementary material for: The C-Terminal Effector Domain of Non-Structural Protein 1 of Influenza A Virus Blocks IFN-β Production by Targeting TNF Receptor-Associated Factor 3
Source: Front Immunol. 2017 Jul 3;8:779. doi: 10.3389/fimmu.2017.00779 (PMC5494602; doi:10.3389/fimmu.2017.00779)

Supplementary Material

The C-Terminal Effector Domain of NS1 Protein of Influenza A Virus blocks IFN-β Production by Targeting TRAF3

Wei Qian[1,2], Xiaoqin Wei[1,2,3], Kelei Guo[1,2], Yongtao Li[4], Xian Lin[1,2], Zhong Zou[1,2], Hongbo Zhou[1,2], Meilin Jin[1,2,5*]

*** Correspondence:**

Meilin Jin
jml8328@126.com

Figure S1. The NS1 proteins of novel H7N9 or avian H9N2 viruses did not interact with TRAF3. 293T cells were cotransfected with plasmid expressing indicated NS1 proteins and Flag-TRAF3 (3 μg each) for 36 h. Cell lysates were harvested, subjected to immunoprecipitation using an anti-Flag antibody, the immunoprecipitates were analyzed by immunoblotting. Expression of the transfected proteins was determined by western blotting using the indicated antibodies (bottom).

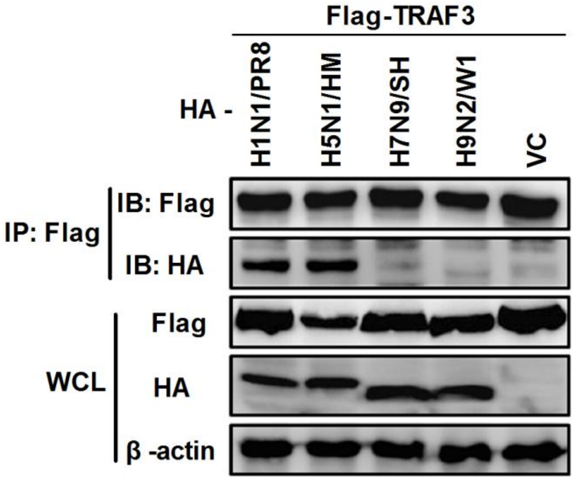

Supplement: Supplementary file 1 [file image_1.pdf]
